# Understanding Mental Health in Autism: A Lifespan Perspective

**DOI:** 10.3390/brainsci15070774

**Published:** 2025-07-21

**Authors:** Domingo García-Villamisar, María Álvarez-Couto, Gema Pilar Sáez-Suanes

**Affiliations:** 1 Department of Personality, Evaluation and Clinical Psychology, Complutense University of Madrid, 28040 Madrid, Spain; villamis@edu.ucm.es; 2 Department of Education, Research Methods and Evaluation, Faculty of Human and Social Sciences, Comillas Pontifical University, 28049 Madrid, Spain; 3 Department of Educational and Developmental Psychology, Faculty of Teacher Training and Education, Autonomous University of Madrid, 28049 Madrid, Spain; gemap.saez@uam.es

Understanding and studying the mental health of people with ASD across the spectrum, from childhood to adulthood, is undoubtedly a pressing need and a challenge. It is necessary because ASD is a developmental condition that rarely occurs in isolation; in fact, studies indicate that up to 74% of individuals with ASD have at least one additional comorbid diagnosis [1]. Among the most common are intellectual disability—ID [2,3], attention deficit and hyperactivity disorder—ADHD [4], or anxiety [5]. Mental health consequences are evident not only for individuals with ASD, but also for their families and close relations.

At the same time, it is a challenge due to the immense heterogeneity in the manifestations and characteristics of ASD [6], which makes it extremely difficult to identify patterns and models of psychopathology and mental health that apply to the entire population.

Therefore, this Special Issue addresses various aspects related, directly or indirectly, to mental health throughout different stages of life and in different domains. It includes studies related to identification and assessment, effective interventions and specialized care models, as well as the intersections between mental health and other aspects of life for individuals with autism, such as quality of life, social inclusion, or family experiences.

Across the 18 contributions that make up this Special Issue, readers can explore and reflect on some of these aspects and their related variables at various stages of development. Thirteen of the articles are research papers covering a range of topics that can be grouped into five main themes:

- Broadening the understanding of ASD by considering its heterogeneity through a proposed representation model (Portolese et al., Contribution 13);
- Transdiagnostic variables related to various psychopathological traits—including Kouklari et al. (Contribution 18), who study executive functions in relation to schizotypal traits in children aged 7 to 12 with ASD, and Recio et al. (Contribution 6), who analyze the relationship between worry, the intolerance of uncertainty, and stress in autistic adults aged 24 to 46;
- Understanding comorbidity in people with ASD, either in general—such as the study by Cagiano et al. (Contribution 17) on comorbidity in adolescents with ASD without intellectual disability (ID)—or related to specific disorders. Noteworthy here are studies on anxiety, such as those by Bitsika et al. (Contribution 15) and Hildebrandt et al. (Contribution 14) focusing on youth with ASD, and that of Rodríguez-Jiménez and Martínez-González (Contribution 3) on school-aged children. This theme also includes studies analyzing obsessions related to maladaptive behaviors (Carpita et al., Contribution 10), and the relationship between ASD and ADHD (Fusar-Poli et al., Contribution 2);
- Intervention proposals addressing socioemotional behaviors, including the proposal by Scuotto et al. (Contribution 12) of an emotional recognition task for adults, and LEGO^®^ therapy to improve social behavior in children without ID by Oliveira et al. (Contribution 7);
- Analysis of schooling and school participation, with proposals by Villegas and Codina (Contribution 9) and Pietragalla et al. (Contribution 1).

The remaining five articles are literature reviews on various topics:

- Comorbidity: either regarding specific disorders, such as the proposal by Tateno et al. (Contribution 11) on gender dysphoria, or studying general mental health. Sánchez and Luque (Contributions 8 and 16) focus on the mental health impact of COVID-19 on individuals with ASD and their families, while Tafolla and Lord (Contribution 5) provide a longitudinal perspective.
- Effects and benefits of mindfulness-based interventions aimed at individuals with ASD (Simione et al., Contribution 4).

## Conclusions

This Special Issue highlights the growing awareness and need to gain a deeper understanding of the mental health of individuals on the autism spectrum throughout their lives. Through empirical studies, systematic reviews, instrument validations, and qualitative analyses, the authors offer diverse and rich perspectives on the factors influencing emotional well-being, subjective experiences, and the social contexts that shape mental health in autism.

Collectively, these studies underscore the urgency of developing interventions that are not only clinically effective, but also aligned with the priorities expressed by autistic individuals and their families. Future research should continue to integrate participatory methodologies, neurodiverse perspectives, and an intersectional lens that can address the complexity of autistic experiences in relation to mental health.

This Special Issue aspires to serve as a resource for researchers, educators, and practitioners, aiming to provide valuable knowledge and practical guidance to improve the quality of life and emotional well-being of individuals with autism.

We thank all the authors and reviewers for their contributions and dedication in making this Special Issue possible. We invite the scientific and professional community to explore these articles and continue the dialog on mental health in autism.
